# Functional studies of signaling pathways in peri-implantation development of the mouse embryo by RNAi

**DOI:** 10.1186/1471-213X-5-28

**Published:** 2005-12-28

**Authors:** Miguel L Soares, Seiki Haraguchi, Maria-Elena Torres-Padilla, Tibor Kalmar, Lee Carpenter, Graham Bell, Alastair Morrison, Christopher JA Ring, Neil J Clarke, David M Glover, Magdalena Zernicka-Goetz

**Affiliations:** 1 The Wellcome Trust/Cancer Research UK Gurdon Institute, University of Cambridge, Tennis Court Road, Cambridge CB2 1QR, UK; 2 Department of Genetics, University of Cambridge, Downing Street, Cambridge CB2 3EH, UK; 3 Polgen Division, Cyclacel Ltd, Babraham Science Park, Babraham, Cambridge CB4 2AT, UK; 4 GlaxoSmithKline Research and Development, Gunnels Wood Road, Stevenage, SG1 2NY, UK

## Abstract

**Background:**

Studies of gene function in the mouse have relied mainly on gene targeting via homologous recombination. However, this approach is difficult to apply in specific windows of time, and to simultaneously knock-down multiple genes. Here we report an efficient method for dsRNA-mediated gene silencing in late cleavage-stage mouse embryos that permits examination of phenotypes at post-implantation stages.

**Results:**

We show that introduction of Bmp4 dsRNA into intact blastocysts by electroporation recapitulates the genetic *Bmp4 *null phenotype at gastrulation. It also reveals a novel role for Bmp4 in the regulation the anterior visceral endoderm specific gene expression and its positioning. We also show that RNAi can be used to simultaneously target several genes. When applied to the three murine isoforms of Dishevelled, it leads to earlier defects than previously observed in double knock-outs. These include severe delays in post-implantation development and defects in the anterior midline and neural folds at headfold stages.

**Conclusion:**

Our results indicate that the BMP4 signalling pathway contributes to the development of the anterior visceral endoderm, and reveal an early functional redundancy between the products of the murine Dishevelled genes. The proposed approach constitutes a powerful tool to screen the functions of genes that govern the development of the mouse embryo.

## Background

Embryonic patterning in mammalian peri-implantation development is a complex process requiring many signaling events to establish the future body plan. The multiplicity of isoforms of various components of these signaling pathways makes it difficult to use classical knock-out techniques to understand these processes. Another difficulty arises in assessing the roles of genes required in several different windows of developmental time. This is because targeted deletion of genes with essential roles in development often results in early embryonic lethality preventing the evaluation of later functions. The use of the Cre recombinase for temporal- and tissue-specific gene knock-out provides one route to circumvent this problem [1]. Nonetheless, such approaches are both costly and time consuming.

In several organisms the above difficulties have been at least partially addressed by the use of gene silencing by RNA interference (RNAi) [2-4]. A primary obstacle in using RNAi in the mouse is in the delivery of dsRNA molecules into the embryo and subsequent analysis of its development. RNAi has been employed successfully in oocytes and early pre-implantation embryos up to the 4-cell stage by microinjection and electroporation [5-8]. However, its application at later stages could be considered more demanding as from the 8-cell stage onwards, some cells become internalized and therefore less accessible. Electroporation has also been used to deliver dsRNA to post-implantation embryos *ex-utero *[9]. This approach is particularly valuable for regional gene knockdown. However, in some instances it would be useful to have the possibility to interfere with gene expression in the whole embryo. Transgenic expression of siRNA has also been reported in siRNA-electroporated ES cells aggregated with tetraploid blastomeres [10]. This approach is a valuable way of targeting knock-down to the embryo proper, but does not allow targeting of genes expressed in extraembryonic tissues such as the extraembryonic ectoderm (ExE) and the visceral endoderm (VE) that do not arise from the ES cell progeny. As signaling often arises in extra-embryonic tissues, RNAi approaches effective in such tissues could prove very valuable.

We wished to enable studies of the effects of knocking-down single or multiple genes simultaneously upon patterning of the early post-implantation mouse embryo. To this end we have developed a method for gene silencing that allows dsRNA to be introduced into whole embryos at late pre-implantation stages such that they can develop *in utero *and be examined post-implantation (Additional File 1). In developing this method we have examined the consequences of knocking-down Bmp4 expression in specific windows of time. This revealed an unexpected contribution of this signaling pathway to the development of the anterior visceral endoderm. We then applied our method to simultaneously knock-down the three Dishevelled isoforms. Our results demonstrate functional redundancy between the products of these genes that when down-regulated together have earlier effects upon development than previously reported.

**Figure 1:**
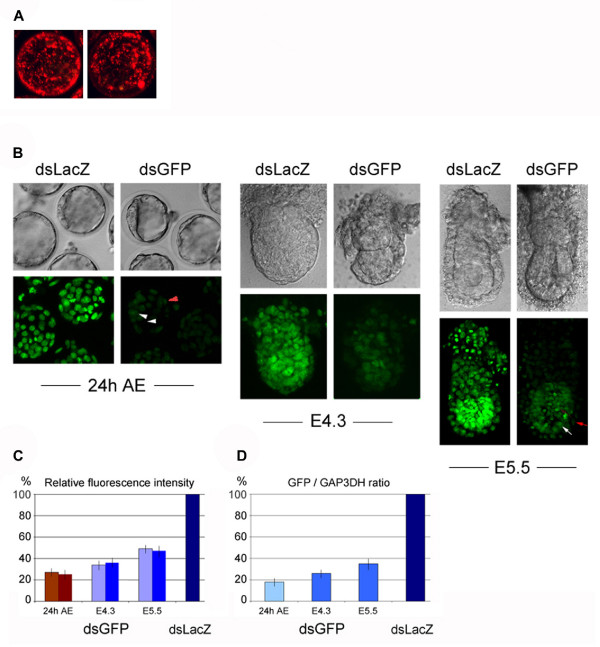
**Effective dsRNA delivery and EGFP knock-down by electroporation. **(A) Representative dark-field projections of the rendered z-stack of x-y confocal sections of blastocysts electroporated with AF594-labelled dsRNA (540 bp) after 24 h of culture. One hundred percent of the blastocysts incorporate the dsRNA and an average of 90% of the blastomeres in each embryo (minimum 70%) are targeted. Left, the ICM is viewed from the top; right, the ICM is viewed laterally. (B) Representative confocal bright-field images (top) and dark-field projections of the rendered z-stack of x-y sections (bottom) of HB2-EGFP embryos electroporated with GFP dsRNA and LacZ dsRNA (control) at the 8-cell and blastocyst stages, followed by 24 hours in culture (24 AE) and *in utero *development until the indicated stages (E4.3 and E5.5). The reduction of GFP fluorescence intensity following RNAi was similar among both the ICM cells (white arrowheads) and trophectoderm cells (red arrowhead). Likewise, despite the difference in fluorescence intensity between the epiblast cells and the surrounding extraembryonic tissues, GFP fluorescence was reduced to a similar degree in both tissues (white arrow and red arrow, respectively). (C) Average levels of fluorescence intensity of the main cell lineages of GFP dsRNA-electroporated blastocysts (24 h AE), and post-implantation embryos at the indicated stages, shown as a percentage of the intensity levels of control (LacZ dsRNA-treated) embryos. Bar colors as follows: cultured blastocysts (n = 9): brown, ICM; dark red, trophectoderm; E4.3 and E5.5 embryos (n = 8 and n = 7, respectively): blue, epiblast; light blue, visceral endoderm and extraembryonic ectoderm. Note that the reduction of fluorescence intensity among the different lineages at any given stage of development is very similar. Standard deviation bars are indicated. (D) Average GFP mRNA levels (GFP/GAP3DH ratio) of embryos electroporated with GFP dsRNA shown as a percentage of those of LacZ dsRNA-treated controls. Pale blue, 15 dsGFP-electroporated embryos cultured *in vitro *for 24 h (control embryos: 15, dark blue). Light blue, 4 and 3 dsGFP-electroporated embryos recovered at E4.3 and E5.5, respectively, following *in utero *development (control embryos: 8, dark blue). Standard deviation bars are indicated.

## Results and Discussion

### Effective EGFP knock-down by dsRNA electroporation

We first determined the most efficient conditions for the uptake of dsRNA between the 8-cell and blastocyst stages using labeled GFP dsRNA (540 bp) and were able to develop parameters for electroporation of dsRNA that did not require thinning of the *zona pellucida *(see Methods). Our final method permitted uptake of dsRNA into 100% of the electroporated embryos (n = 120). Optical sectioning by confocal microscopy at the blastocyst stage indicated that greater than 90% of the cells in each embryo were targeted by the labeled dsRNA on average (minimum 70%) (Fig. 1A).

To assess the relative efficiency of knock-down in the different cell lineages at both pre- and post-implantation stages, we used a transgenic mouse line expressing the fluorescent fusion reporter histone (H2B)-EGFP [11]. Intact embryos carrying the H2B-EGFP transgene, which is ubiquitously expressed in a high-level constitutive manner, were electroporated with GFP dsRNA (and LacZ dsRNA, as a control) at the early 8-cell and blastocyst stages. Confocal fluorescence microscopy following *in vitro *culture for 24 h showed that the delivery of the GFP dsRNA had been equally effective in reducing the levels of H2B-EGFP fluorescence in both trophectoderm and ICM lineages (Fig 1B,C). Quantification of the fluorescence intensity revealed a 73% overall reduction of fluorescence in the GFP dsRNA-treated embryos compared to the LacZ dsRNA-electroporated controls (Fig. 1C). The relative reduction of the fluorescence levels in the ICM and trophectoderm cells was calculated to be of similar degree. The overall reduction of H2B-GFP expression correlates well with the strong knock-down at the mRNA level (80%) as determined by RT-PCR (Fig. 1D).

Electroporated embryos were also transferred into the uteri of pseudopregnant females and recovered and analyzed post-implantation at E4.3 and E5.5. As before, the levels of histone-GFP fluorescence were much reduced in GFP dsRNA-treated embryos and to a similar extent in the different lineages of the pre-streak stage embryo. The epiblast, although of overall higher GFP fluorescence intensity than the surrounding lineages, had its relative fluorescence reduced to the same degree as the VE and ExE lineages: an average 65% and 52% reduction at E4.3 and E5.5, respectively (Fig. 1B,C). The GFP mRNA levels were still strongly depleted at these stages despite the differences of overall fluorescence intensity being less pronounced between the GFP dsRNA-treated embryos and controls (Fig 1D). This could be explained by the stability of the EGFP fusion protein, which remains bound to the target even during cell division when the nuclear envelope has broken down [11]. The reduction of H2B-GFP fluorescence levels induced by GFP dsRNA electroporation was actually surprisingly high for a highly expressed protein product that starts being expressed at the 4-cell stage and, because of its inherent stability, persists in the cells for long periods of time.

### *Bmp4 *knock-down recapitulates null phenotype and reveals new Bmp4 function

To further study the role of key signaling pathways in peri-implantation development, we chose to target the endogenous mouse gene *Bmp4*, a member of the TGF-β superfamily of secreted signaling molecules [12]. We wished to determine whether electroporation of pre-implantation embryos with Bmp4 dsRNA led to similar phenotypes, specifically in events around the time of gastrulation, as reported for the genetic loss of its function [13-15]. *Bmp4 *is first expressed in the ICM and polar trophectoderm of the embryonic day 3.5 (E3.5) blastocyst. As the embryo develops to E6.5 its expression becomes restricted to the ExE and is highest in the cells abutting the epiblast close to the boundary between the embryonic and extraembryonic regions of the conceptus [16]. After gastrulation, *Bmp4 *is expressed in epiblast-derived tissues, including the extraembryonic mesoderm [15].

**Figure 2:**
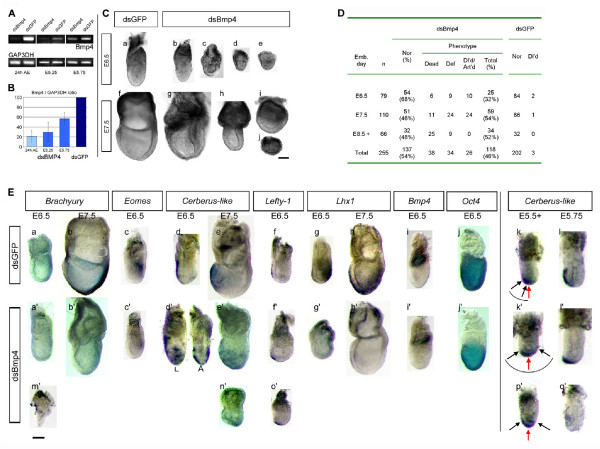
**Specific *Bmp4 *knock-down by RNAi induces a range of developmental defects post-implantation. **(A) RT-PCR of single embryos cultured *in vitro *for 24 h following electroporation, and embryos recovered at E5.25 and E5.75 following development *in utero*. Glyceraldehyde 3-phosphate dehydrogenase (GAP3DH) was used as an internal control for cDNA loading. (B) Average Bmp4 mRNA levels (Bmp4/GAP3DH ratio) of embryos electroporated with Bmp4 dsRNA (dsBmp4) shown as a percentage of those of GFP dsRNA (dsGFP)-treated controls. Pale blue, 20 dsBmp4-electroporated embryos cultured *in vitro *for 24 h (control embryos: 20, dark blue). Light blue, 10 dsBmp4-electroporated embryos recovered at E5.25 and E5.75, respectively, following *in utero *development (control embryos: 10). Standard deviation bars are indicated. (C) Morphological phenotypes of dsBmp4 (B-E, G-J) and control dsGFP -electroporated embryos (a, f) recovered at E6.5 and E7.5 (DIC micrographs). Anterior faces left when it can be identified. E6.5 and E7.5 represent the embryonic time at collection; actual embryonic stages may vary due to Bmp4 RNAi. dsBmp4-treated embryos are generally reduced size and acquire an abnormal round shape (c-e, i, j); or present gross abnormalities in (g) or lack recognizable embryonic structures (j). Some embryos show an underdeveloped epiblast relative to the ExE (h). The well developed ExM-derived allantois as seen in the control (f) is not normally observed in dsBmp4-treated embryos. Bar, 200 μm. (D) Distribution of morphological phenotypes observed past gastrulation. Emb. day, embryonic day; Nor, morphologically normal; Def, defective; Dl'd, delayed, Art'd, arrested. (E) Whole-mount *in situ *hybridization with anti-sense probes of indicated genes carried out on control dsGFP RNA (a-l) and dsBmp4 RNA -electroporated embryos (a'-q'). The developmental stages shown represent the embryonic time at collection; actual embryonic stages may vary due to *Bmp4 *RNAi. Over 50% of the dsBmp4-electroporated embryos [developmentally delayed but otherwise normal and morphologically normal (group1)] express much reduced amounts of mesodermal *Brachyury *(E6.5 n = 8/15; E7.5 n = 8/16), *Eomes *(E6.5 n = 5/9; E7.5 n = 4/7) and *Lhx1 *(E6.5 n = 3/5; E7.5 n = 3/6), suggesting that gastrulation was initiated but failed to continue further (a'-c', g'-h'). In contrast, all defective embryos (group2) lack expression of these genes altogether (E6.5 n = 2, 2, 1; E7.5 n = 3, 3, 2, respectively), indicating that they failed to gastrulate (m'). The expression of visceral endoderm *Cer-l*, *Lefty-1 *and *Lhx1 *markers was also abnormal in the dsBmp4-treated embryos. While *Lhx1 *expression was strongly downregulated in the VE of 55% of group 1 embryos (g', h') (E6.5 n = 3/5; E7.5 n = 3/6), it was completely absent in defective embryos (n = 3, not shown). Similarly, *Lefty-1 *transcripts were absent (E6.5 n = 1; E7.5 n = 2, not shown) or restricted to the DVE (E6.5 n = 1; E7.5 n = 2) in defective embryos (O'). In 55% of group 1 embryos (n = 11), *Lefty-1 *expression was found at or close to the DVE (f') (E6.5 n = 3/6; E7.5 n = 3/5). Accordingly, the expression of *Cer-l *was also found to be restricted to the DVE in all defective embryos essayed (n') (n = 5). In over 50% (16/31) of the embryos of group 1, the expression was either distally restricted and/or mislocalized, with ectopic transcripts scattered around the embryonic region in the lateral VE at both E6.5 (d') (n = 9) and E7.5 (e') (n = 7), denoting a lack of migration of the DVE cells and ectopic expression of *Cer-l*. As early as E5.5 and E5.75 the *Cer-l *expression domain was extended towards both anterior and posterior regions of the VE overlying the epiblast (k', p') (n = 8); restricted distally (l') (n = 2) or absent q' (n = 3) in a total of 16 embryos. Red arrows show the midline of the anterior-posterior axis of the embryo; black arrows delimitate the boundaries of *Cer-l *expression in the VE. *Bmp4 *expression in the ExE was already detectable by E6.5 in dsBmp4-electroporated embryos (i'). Epiblast *Oct4 *expression was normal at both E6.5 (j') and E7.5 (not shown). Anterior faces left when it can be identified, except in d' where both lateral (L) and anterior (A) views are presented. Bar, 200 μm.

We introduced Bmp4 dsRNA into intact E3.5 blastocysts by electroporation and first determined the efficiency of knock-down by measuring the *Bmp4 *expression levels 24 h and 48 h later by RT-PCR. Bmp4 mRNA was reduced in Bmp4 dsRNA-electroporated embryos at both 24 h (Fig 2A,B) and 48 h after electroporation (not shown), compared to the GPF dsRNA-electroporated experimental controls. Levels of *Gap3dh *(Fig 2A) and *Stat3 *mRNA (not shown) were unaffected, suggesting that the Bmp4 dsRNA electroporation resulted in sequence-specific knock-down of *Bmp4 *expression. Electroporated embryos were transferred into the uteri of pseudopregnant females and then recovered and analyzed at several stages post-implantation. *Bmp4 *expression was reduced by as much as 70% at E5.25 and 44% at E5.75, as determined by RT-PCR (Fig 2A,B). The Bmp4 dsRNA-electroporated embryos displayed a range of phenotypes characteristic of that of *Bmp4 *null embryos many of which arrest in development shortly after implantation, but a proportion showing some development to later stages [13]. Between E6.5 and E7.5, and at some later stages, 46% (n = 255) of Bmp4 dsRNA-electroporated embryos were reduced in size by at least 30%. They were also rounded, disorganized or necrotic, and had underdeveloped epiblasts (Fig 2C). This contrasted with the 1% (n = 202) of control embryos that were slightly delayed in development, but morphologically normal, demonstrating that the long dsRNA has a specific effect and that, in accordance to other reports, does not elicit non-specific responses at these stages [7,9]. At E7.5 Bmp4 dsRNA-electroporated embryos also lacked or had a reduced mesoderm-derived allantois (Fig 2C, panel g).

Bmp4 is required for epiblast cell proliferation, mesoderm formation [13,14] and has been suggested to promote differentiation of the VE [16]. We therefore examined the expression of a set of molecular markers of the prospective mesoderm (*Brachyury*, *Eomes*, *Lhx1*) and visceral endoderm (*Cer-l*, *Lefty-1*, *Lhx1*) at E6.5 and E7.5 following Bmp4 RNAi. *Brachyury*, *Eomes *and posterior *Lhx1 *expression was absent in all defective embryos. It was also absent or significantly reduced in over 52% of the remaining group of Bmp4 dsRNA-electroporated embryos that appeared morphologically normal, whether delayed in development or not (Fig 2E, panels a'-c', g', h', and Additional File 1). This reduced expression of mesoderm markers suggests that in some embryos primitive streak formation and the initiation of gastrulation probably occurred but failed to progress further. It is consistent with the reported phenotypes of *Bmp4*^-/- ^embryos [13].

The consequences for the expression of markers of the anterior VE (AVE) were less expected. Before gastrulation, by E5.5, *Cer-l *and *Lefty-1 *transcripts are normally restricted to the distal tip of the VE (DVE). These DVE cells then move towards the prospective anterior of the embryo and so by E6.0 become the AVE that plays a key role in anterior patterning [17,18]. We found that the expression of *Cer-l *was abnormal in Bmp4 dsRNA-electroporated embryos already at E5.5 and E5.75 (Fig 2E and Additional File 1). In a small number of embryos (3/16) we were unable to detect *Cer-l *transcripts at these stages (Fig 2E, panel q'). Rather than being slightly shifted towards the prospective anterior at E5.5 [18], we found that in 50% of Bmp4 dsRNA-treated embryos (n = 8/16), the *Cer-l *expressing domain was expanded symmetrically around the distal tip towards both the anterior and posterior regions of the VE (Fig 2E, panels k', p'). Between E6.5 and E7.5, *Cer-l *expression was either abolished or localized to the distal tip in all morphologically defective embryos. Moreover, in 52% (n = 16/31) of the remaining group of dsBmp4-treated embryos of normal morphology we observed an extensive diffuse region of ectopic *Cer-l *expression around the DVE and in the lateral VE (Fig 2E, panels d'-e'). *Lefty-1 *expression had a similar pattern to that of *Cer-l *in all defective embryos at these stages, as well as in 55% (6/11) of the remaining Bmp4 dsRNA-treated embryos, although no ectopic transcripts were observed (see Additional File 1). *Lhx1*, which was expressed throughout the AVE in the control embryos, was downregulated in 55% of the Bmp4 dsRNA-electroporated embryos (n = 6/11), thus confirming a disruption of AVE patterning.

Thus, our results indicate that down-regulation of *Bmp4 *expression changes the AVE expression domain. The expression of DVE markers either remains distal (*Cer-l *and *Lefty-1*) or spreads laterally (*Cer-l*). This suggests that Bmp4 signals that mainly originate from the ExE at these and earlier stages can regulate the restriction of the AVE expression domain and its specific positioning. This pattern of regulation is in agreement with a report that showed that BMP signaling promotes differentiation of VE in embryoid bodies [16]. It is also consistent with the phenotype of the BMP antagonists *Chordin *and *Noggin *double mutants, which fail to maintain expression of *Cer-l *at E7.5 [19], supporting the role of Bmp4 in regulating *Cer-l*.

Our results also indicate that the effects of Bmp4 in patterning the VE occur in a time window between E3.5 and E5.5. We observed that a proportion of embryos can "recover" from the transient nature of the knock-down during the course of the experiment, as determined by RT-PCR at E5.75 (Fig 2B) and at E6.5 by *in situ *hybridization (Fig 2E, panel i'). The transient nature of the RNAi effect can be seen as an advantage in a given experimental context, as it enables the period of gene expression required for events relating to embryo patterning to be pin-pointed. The definition of time intervals in which essential signaling molecules are required is critical for the understanding of the AVE specification and subsequent migration. Thus the approach we propose here might prove very valuable in addressing AVE function as it allows modulating gene expression just after embryo implantation.

Aside from these novel findings, the other phenotypes following Bmp4RNAi are strikingly similar to those reported for null mutant embryos both in form and variability. Approximately 20% of embryos developed to E9.5, albeit with severe defects [13]. In all, over 70% of the embryos were either morphologically abnormal and/or defective in the expression of essential mesoderm and VE markers following Bmp4 RNAi. It cannot be excluded that some part of the observed pleiotropy may be attributed to differential uptake of Bmp4 dsRNA, although our experiments with the H2B-GFP transgenic embryos indicate that all cell lineages are targeted by the dsRNA to a very similar extent.

### Simultaneous knock-down of the three murine *Dishevelled *genes

**Figure 3:**
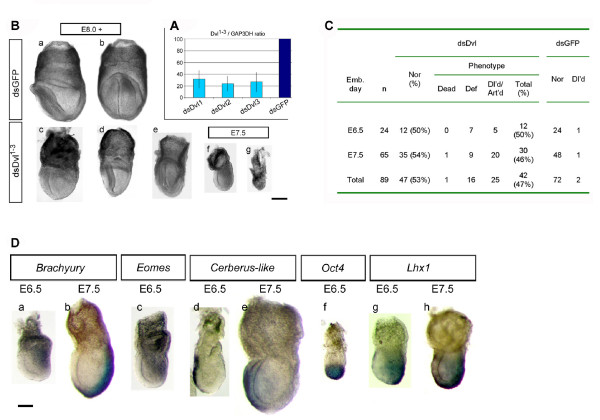
**Specific *Dvl*^1–3 ^knockdown induces developmental delays post-implantation, and anterior midline and neural fold defects. **(A) Average *Dvl*^1–3 ^mRNA levels (Dvl^1–3^/GAP3DH ratio) of 10 embryos electroporated with dsDvl^1–3 ^shown as a percentage of those of 10 dsGFP-treated controls, following 24 h in culture. Standard deviation bars are indicated. (B) Morphological phenotypes of dsDvl^1–3 ^(c-g) and control dsGFP -electroporated embryos (a, b) recovered at E7.5 and E8.0 (brightfield micrographs). Anterior faces left when it can be identified, except in b-e, where an anterior view is presented. E7.5 and E8.0 represent the embryonic time at collection; actual embryonic stages may vary due to *Dvl *RNAi. dsDvl^1–3^-treated embryos are smaller than normal and retarded in development (c-f) (compare with f in Fig 2C, note scale bars). Note the severe delay in f and the early developmental arrest in g. Notably, in some E8.0 embryos, the anterior midline was grossly distorted and, in some cases, was connected anteriorly to a single neural fold of either side (d, e), giving to the anterior view the appearance of a slightly-lateralized view. Bar, 200 μm. (C) Distribution of morphological phenotypes observed past gastrulation. Emb. day, embryonic day; Nor, normal; Def, defective; Dl'd, delayed, Art'd, arrested. (D) Whole-mount in situ hybridization with anti-sense probes of indicated genes on dsDvl^1–3^-electroporated embryos. Anterior faces left. Expression of *Brachyury *(n = 10), *Eomes *(n = 8), *Lhx1 *(n = 8), *Cer-l *(n = 11) and *Oct4 *(n = 6) is seemingly unaffected in these embryos (compare to Fig 2e, top row). Note the severe developmental delay, particularly evident in b, f and h, and the abnormal extraembryonic region in h. Bar, 200 μm.

We next wished to determine whether this approach could enable functional redundancy between genes to be examined. To this end, we simultaneously targeted the three murine *Dishevelled *(*Dvl*^1–3^) genes [20-22]. The Dishevelled proteins are members of the Wnt developmental pathway, which regulates cell fate and subsequent cell behaviour in metazoans [23]. Analysis of the genetic null mutants (single and double mutants) has suggested redundancy of function among these genes [24-26]. To our knowledge a triple genetic knockout of these genes has not been reported, perhaps due to the difficulty of generating such a mutant. Although the three isoforms are expressed throughout pre-implantation development, a significant increase in expression is seen from the morula stage onwards, which is shared by a number of Wnt family members [27]. Thus, to ensure effective knock-down of all three *Dvl *genes we subjected embryos to co-electroporation with the corresponding three dsRNAs at two successive stages. The first treatment was given to 8-cell stage embryos and was followed by a second treatment when the embryos had developed to the blastocyst stage. A significant knock-down of the *Dvl *mRNA levels (average 70% *Dvl1*, 76% *Dvl2 *and 73% *Dvl3*) was observed 24 h (Fig 3A) and 48 h (not shown) after each electroporation treatment. We also electroporated each Dvl dsRNA independently and found that the mRNA knock-down achieved was similar to that observed upon co-electroporation of the three *Dvl *simultaneously (n = 10 for each *Dvl*).

Following a period of development *in utero*, the embryos were recovered and analyzed at E6.5 and E7.5 or E8.0. While the embryos treated with a single Dvl dsRNA did not shown any morphological abnormalities at these stages (for each *Dvl*: n = 14, E6.5 and n = 15, E7.5^+^), nearly half (42/89) of the Dvl^1–3 ^dsRNA-electroporated embryos were severely retarded in development or showed morphological abnormalities (Fig 3B,C). Forty percent of the affected embryos had not gastrulated as assessed by morphological analyses (Fig. 3B, panel g). In contrast, only 2 of 72 dsGFP-electroporated control embryos were developmentally delayed but showed no apparent defects. Interestingly, approximately 12% (n = 65) of the Dvl^1–3 ^dsRNA-treated embryos examined at E8.0 showed a grossly distorted anterior midline and neural fold abnormalities. These were developmentally retarded and had a sinuous or bent anterior axial mesendoderm, which seemed to be connected anteriorly to a single neural fold of either side (Fig 3B, panel c-e). These defects appear to represent an earlier onset of the later neural tube closure phenotype observed in *Dvl1*^-/-^; *Dvl2*^-/- ^double mutants [25]. Unexpectedly, neither the expression of mesodermal markers *Brachyury*, *Eomes*, and *Lhx1*, the VE markers *Cer-l *and *Lhx1*, nor the epiblast marker *Oct4 *appeared to be abnormal in a set of developmentally delayed embryos following dsDvl^1–3 ^dsRNAi (Fig 3D). This indicates that while the knock-down of *Dvl *was effective in the "delayed" embryos – and enough to cause developmental delays – it was not sufficient to prevent gastrulation, such as observed for the group of morphologically defective embryos. The failure to gastrulate observed in the latter set of embryos upon Dvl^1–3 ^RNAi is in agreement with the role of the Wnt signaling pathway in primitive streak formation, as typified by the *Wnt3 *mutants and *Lrp5*^-/-^;*Lrp6*^-/- ^double mutants [28,29].

It is possible that the observed pleiotropy of the phenotype following *Dvl *down-regulation can be attributed to differential uptake of dsRNA. Based on the relative kinetics of EGFP and *Bmp4 *knock-down, it seems likely that the efficacy of knock-down and the rate of mRNA recovery are target dependent. Thus, factors such as the level of expression, turnover rate of mRNA species, and protein product stability/degradation might dictate the extent of the RNAi phenotype.

Our results have shown novel phenotypes following down-regulation of specific genes and so demonstrate that electroporation of pre-implantation embryos with single or multiple dsRNAs directed against genes encoding related proteins is effective.

## Conclusion

In this paper we describe a novel function for *Bmp4 *in regulating the development of the anterior-posterior axis: *Bmp4 *knock-down perturbs the specific positioning of the AVE and may induce ectopic expression of *Cer-l *in the lateral and posterior VE of the embryo. The simultaneous knock-down of all three *Dvl *isoforms leads to earlier defects in development than those reported for double knockout mutants. This indicates an early functional redundancy between these genes. The method we describe here enables the functional study of single or multiple genes within a specific window of developmental time. It offers a relatively rapid approach to assay gene function in the peri-implantation period of development.

## Methods

### dsRNA preparation

RNAs were *in vitro *transcribed from PCR-generated templates using the Ribomax Large-Scale RNA Production System (Promega, Southampton, UK). Chimeric primers containing the T7 promoter sequence and gene-specific sequences were used to amplify *Bmp4 *(nt 2–601, 599 bp), *Dvl1 *(nt 960–1406, 446 bp), *Dvl2 *(nt 864–1393, 547 bp), *Dvl3 *(nt 1479-1061, 418 bp), *GFP *(nt 98–638, 540 bp), and LacZ (521 bp) templates. *In vitro *transcription reactions and subsequent DNAse treatment and RNA purification were performed according to the manufacturer's instructions. The purified RNA was denatured at 70°C for 10 min and let anneal at RT for several hours. The quality and concentration of dsRNA were determined by gel electrophoresis and spectrophotometry. For electroporation, dsRNA was diluted in chilled hepes buffered saline (HBS) to a concentration of 2 mg/ml (dsBmp4, dsDvl1, dsDvl2, dsDvl3, dsGFP and dsLacZ) or 6 mg/ml (dsGFP, as a control for the co-electroporation of the three dsDvl).

### dsRNA labelling

RNAs were *in vitro *transcribed as described above in a 100 μl reaction according to the manufacturer instructions but with 75 mM aminoallyl-UTP (Sigma). Following annealing, the dsRNA was passed through BioRad P30 MicroBiospin columns (BioRad, Hemel Hempstead, UK) previously buffer-exchanged to 100 mM NaHCO_3 _(pH7.5), and mixed at RT, dark O/N with 33 μl of 10 mg/ml Alexa Fluor 594-succinimidyl ester (Molecular Probes) in DMSO. After phenol/chloroform extraction and isopropanol precipitation, the dsRNA was further purified through BioRad P30 MicroBiospin columns (10 mM Tris pH7.4) and an additional phenol/chloroform and isopropanol step.

### Embryo collection and culture

All experimental procedures with live animals were conducted in accordance with UK Government Home Office Licensing regulations. Embryos were collected from F1 (C57BL/6 × CBA) superovulated females crossed with F1 males or transgenic histone H2B-GFP males [11]. Superovulation was carried out by intraperitoneal injection of 10 IU of pregnant mare serum gonadotrophin (PMSG, Folligon, Intervet) followed by 10 IU of human chorionic gonadotrophin (hCG, Corulon, Intervet) 46–48 h later. Fertilized embryos were collected 64–68 h (8-cell stage), 76–78 h (16-cell stage) and 88–90 h (blastocysts) post hCG injection. Collection was performed in M2 medium containing 4 mg/ml bovine serum albumin (BSA) and culture in KSOM supplemented with 4 mg/ml BSA, at 37°C under 5% CO_2_.

### Electroporation and uterine transfer

The embryos were electroporated in a flat electrode chamber (1 mm gap between electrodes) (BTX inc., San Diego, CA) in 50 μl of dsRNA solution in HBS. Electric pulses were delivered using a 2001 Electro Cell Manipulator (BTX inc., San Diego, CA) as follows: 3 sets of 4 pulses of 1 ms at 28–30 V, with 1 minute interval between sets and inverting polarity. Following electroporation, the embryos were cultured in KSOM for subsequent uterine transfer or RT-PCR. After a minimum of 2 hours in culture, the embryos were transferred into pseudo-pregnant F1 females, which had been mated 2.5 days before with vasectomized F1 males. Five embryos were introduced per uterus.

### RT-PCR

One to ten embryos were washed in PBS and lysed in TRIzol (Invitrogen, Paisley, UK) 24 h and 48 h after electroporation. Total RNA was isolated and retro-transcribed by standard procedures. PCR amplification was performed with the following primers: Bmp4 (5'-TGGACTGTTATTATGCCTT-3'; 5'-GGAGATCACCTCATTTTCTGG-3'); Dvl1 (5'-CATCCTCCTTCAGCAGCATCAC-3'; 5'-ACTCGTACCATAGCGGGGC-3'); Dvl2 (5'-AGACTCGGATGAGGATGACA-3'; 5'-AAGGCTCCAGTCAGCGCA-3'); Dvl3 (5'-TGGGGCTGTGGCAGCTGATGG-3'; 5'-GAGGCCATGGCTTTTACGATG); GFP (5'-ACGTAAACGGCCACAAGTTC-3'; 5'-GTTGGGGTCTTTGCTCAGG-3'); and GAPDH (5'-GCATGGACTGTGGTCATGAG-3'; 5'-CCATCACCATCTTCCAGGAG-3'). For each gene, the PCR parameters were optimized to detect differences in mRNA levels: *Bmp4 *(39 cycles E3.5, 43 cycles E5.25/E5.75, 60°C annealing), *Dvl1 *(42 cycles, 55°C annealing), *Dvl2 *(38 cycles, 55°C annealing), *Dvl3 *(46 cycles, 60°C annealing), GFP (32 cycles, 60°C annealing) and *GAP3DH *(27 cycles, 60°C annealing). PCR products were electrophoresed in 2.0% agarose gels with ethidium bromide, photographed under UV light and quantified using The Discovery Series Quantity One 1-D Analysis Software Version 4.4.1 (BioRad).

### Whole-mount *in situ *hybridization

Embryos recovered at E5.5, E5.75, E6.5 and E7.5 and dissected free of maternal tissues and Reichert's membrane were fixed in 4% paraformaldehyde in PBS for at least 24 h. Noon on the day of plug is E0.5. Whole mount in situ hybridization was performed as described . Control and experimental embryos were processed simultaneously and developed for the same time length.

### Imaging

For confocal imaging, the embryos were placed between two coverslips with a petroleum jelly seal in droplets of M2 medium (blastocysts) or DMEM medium supplemented with 45% human cord serum (post-implantation embryos). To increase adhesion, slides were previously treated with poly-L-lysine (Sigma) for 5 minutes.

Laser scanning confocal data was taken using 20× air objective lenses on an inverted Nikon microscope with a BioRad MCR scanning head. Images were acquired as *z*-stacks comprising sequential *x*-*y *sections taken at 2–4 μm *z*-intervals. Raw data was processed and quantified using Volocity (Improvision).

## Competing interests

The author(s) declare that they have no competing interests.

## Authors' contributions

MLS designed and carried out most of the experimental work, and drafted the manuscript; SH assisted in experimental design and participated significantly in most experiments; METP performed part of post-implantation embryo recovery and assisted in confocal microscopy; TK and LC assisted in molecular analyses, experimental design and interpretation of data; GB, AM, and CJAR assisted in analysis and interpretation of results; NJC, DMG and MZG coordinated the project; MZG conceived and supervised the project; MLS prepared the manuscript with MZG and DMG. All authors read and approved the final manuscript.

## Supplementary Material

Additional File 1 **Figure 1. Method for RNAi in peri-implantation development**. Schematic representation of the proposed method for transient loss of gene function by electroporation followed by uterine transfer and development *in utero*. Approaches for the assessment of gene-specific knock-down and phenotypic analyses are suggested. **Table 1. Proportion of dsBmp4 RNA-electroporated embryos with defective expression of indicated marker genes**. Group 1, embryos morphologically normal (MN) and developmentally delayed or arrested (DA); group 2, morphologically defective embryos (Df). SExp, symmetrically expanded expression around the distal tip; DR, distally (DVE) restricted expression; Abs, absence of expression. Click here for file

## References

[B1] Nagy A (2000). Cre recombinase: the universal reagent for genome tailoring. Genesis.

[B2] Kamath RS (2003). Systematic functional analysis of the Caenorhabditis elegans genome using RNAi. Nature.

[B3] Hannon GJ (2002). RNA interference. Nature.

[B4] Gunsalus KC, Piano F (2005). RNAi as a tool to study cell biology: building the genome-phenome bridge. Curr Opin Cell Biol.

[B5] Wianny F, Zernicka-Goetz M (2000). Specific interference with gene function by double-stranded RNA in early mouse development. Nat Cell Biol.

[B6] Svoboda P, Stein P, Hayashi H, Schultz RM (2000). Selective reduction of dormant maternal mRNAs in mouse oocytes by RNA interference. Development.

[B7] Grabarek JB, Plusa B, Glover DM, Zernicka-Goetz M (2002). Efficient delivery of dsRNA into zona-enclosed mouse oocytes and preimplantation embryos by electroporation. Genesis.

[B8] Plusa B, Frankenberg S, Chalmers A, Hadjantonakis AK, Moore CA, Papalopulu N, Papaioannou VE, Glover DM, Zernicka-Goetz M (2005). Downregulation of Par3 and aPKC function directs cells towards the ICM in the preimplantation mouse embryo. J Cell Sci.

[B9] Mellitzer G, Hallonet M, Chen L, Ang SL (2002). Spatial and temporal 'knock down' of gene expression by electroporation of double-stranded RNA and morpholinos into early postimplantation mouse embryos. Mech Dev.

[B10] Kunath T, Gish G, Lickert H, Jones N, Pawson T, Rossant J (2003). Transgenic RNA interference in ES cell-derived embryos recapitulates a genetic null phenotype. Nat Biotechnol.

[B11] Hadjantonakis AK, Papaioannou VE (2004). Dynamic in vivo imaging and cell tracking using a histone fluorescent protein fusion in mice. BMC Biotechnol.

[B12] Hogan BL (1996). Bone morphogenetic proteins: multifunctional regulators of vertebrate development. Genes Dev.

[B13] Winnier G, Blessing M, Labosky PA, Hogan BL (1995). Bone morphogenetic protein-4 is required for mesoderm formation and patterning in the mouse. Genes Dev.

[B14] Lawson KA, Dunn NR, Roelen BA, Zeinstra LM, Davis AM, Wright CV, Korving JP, Hogan BL (1999). Bmp4 is required for the generation of primordial germ cells in the mouse embryo. Genes Dev.

[B15] Fujiwara T, Dunn NR, Hogan BL (2001). Bone morphogenetic protein 4 in the extraembryonic mesoderm is required for allantois development and the localization and survival of primordial germ cells in the mouse. Proc Natl Acad Sci USA.

[B16] Coucouvanis E, Martin GR (1999). BMP signaling plays a role in visceral endoderm differentiation and cavitation in the early mouse embryo. Development.

[B17] Perea-Gomez A, Camus A, Moreau A, Grieve K, Moneron G, Dubois A, Cibert C, Collignon J (2004). Initiation of gastrulation in the mouse embryo is preceded by an apparent shift in the orientation of the anterior-posterior axis. Curr Biol.

[B18] Yamamoto M, Saijoh Y, Perea-Gomez A, Shawlot W, Behringer RR, Ang SL, Hamada H, Meno C (2004). Nodal antagonists regulate formation of the anteroposterior axis of the mouse embryo. Nature.

[B19] Bachiller D, Klingensmith J, Kemp C, Belo JA, Anderson RM, May SR, McMahon JA, McMahon AP, Harland RM, Rossant J, De Robertis EM (2000). The organizer factors Chordin and Noggin are required for mouse forebrain development. Nature.

[B20] Sussman DJ, Klingensmith J, Salinas P, Adams PS, Nusse R, Perrimon N (1994). Isolation and characterization of a mouse homolog of the Drosophila segment polarity gene dishevelled. Dev Biol.

[B21] Klingensmith J, Yang Y, Axelrod JD, Beier DR, Perrimon N, Sussman DJ (1996). Conservation of dishevelled structure and function between flies and mice: isolation and characterization of *Dvl2*. Mech Dev.

[B22] Tsang M, Lijam N, Yang Y, Beier DR, Wynshaw-Boris A, Sussman DJ (1996). Isolation and characterization of mouse *Dishevlled-3*. Dev Dyn.

[B23] Moon RT, Brown JD, Torres M (1997). WNTs modulate cell fate and behavior during vertebrate development. Trends Genet.

[B24] Lijam N, Paylor R, McDonald MP, Crawley JN, Deng CX, Herrup K, Stevens KE, Maccaferri G, McBain CJ, Sussman DJ, Wynshaw-Boris A (1997). Social interaction and sensorimotor gating abnormalities in mice lacking Dvl1. Cell.

[B25] Hamblet NS, Lijam N, Ruiz-Lozano P, Wang J, Yang Y, Luo Z, Mei L, Chien KR, Sussman DJ, Wynshaw-Boris A (2002). Dishevelled 2 is essential for cardiac outflow tract development, somite segmentation and neural tube closure. Development.

[B26] Wang J, Hamblet NS, Lijam N, Sussman DJ, Wynshaw-Boris A (2004). The murine *Dishevelled 2 *gene is essential for neural tube closure [abstract]. Mouse Genetics Meeting.

[B27] Wang QT, Piotrowska K, Ciemerych MA, Milenkovic L, Scott MP, Davis RW, Zernicka-Goetz M (2004). A genome-wide study of gene activity reveals developmental signaling pathways in the preimplantation mouse embryo. Dev Cell.

[B28] Liu P, Wakamiya M, Shea MJ, Albrecht U, Behringer RR, Bradley A (1999). Requirement for Wnt3 in vertebrate axis formation. Nat Genet.

[B29] Kelly OG, Pinson KI, Skarnes WC (2004). The Wnt co-receptors Lrp5 and Lrp6 are essential for gastrulation in mice. Development.

