# Supplementary material for: Functional studies of signaling pathways in peri-implantation development of the mouse embryo by RNAi
Source: BMC Dev Biol. 2005 Dec 28;5:28. doi: 10.1186/1471-213X-5-28 (PMC1363358; doi:10.1186/1471-213X-5-28)
Supplement: Additional File 1 — Figure 1. Method for RNAi in peri-implantation development. Schematic representation of the proposed method for transient loss of gene function by electroporation followed by uterine transfer and development in utero. Approaches for the assessment of gene-specific knock-down and phenotypic analyses are suggested. Table 1. Proportion of dsBmp4 RNA-electroporated embryos with defective expression of indicated marker genes. Group 1, embryos morphologically normal (MN) and developmentally delayed or arrested (DA); group 2, morphologically defective embryos (Df). SExp, symmetrically expanded expression around the distal tip; DR, distally (DVE) restricted expression; Abs, absence of expression. [file 1471-213X-5-28-S1.pdf]

Supplementary Figure 1

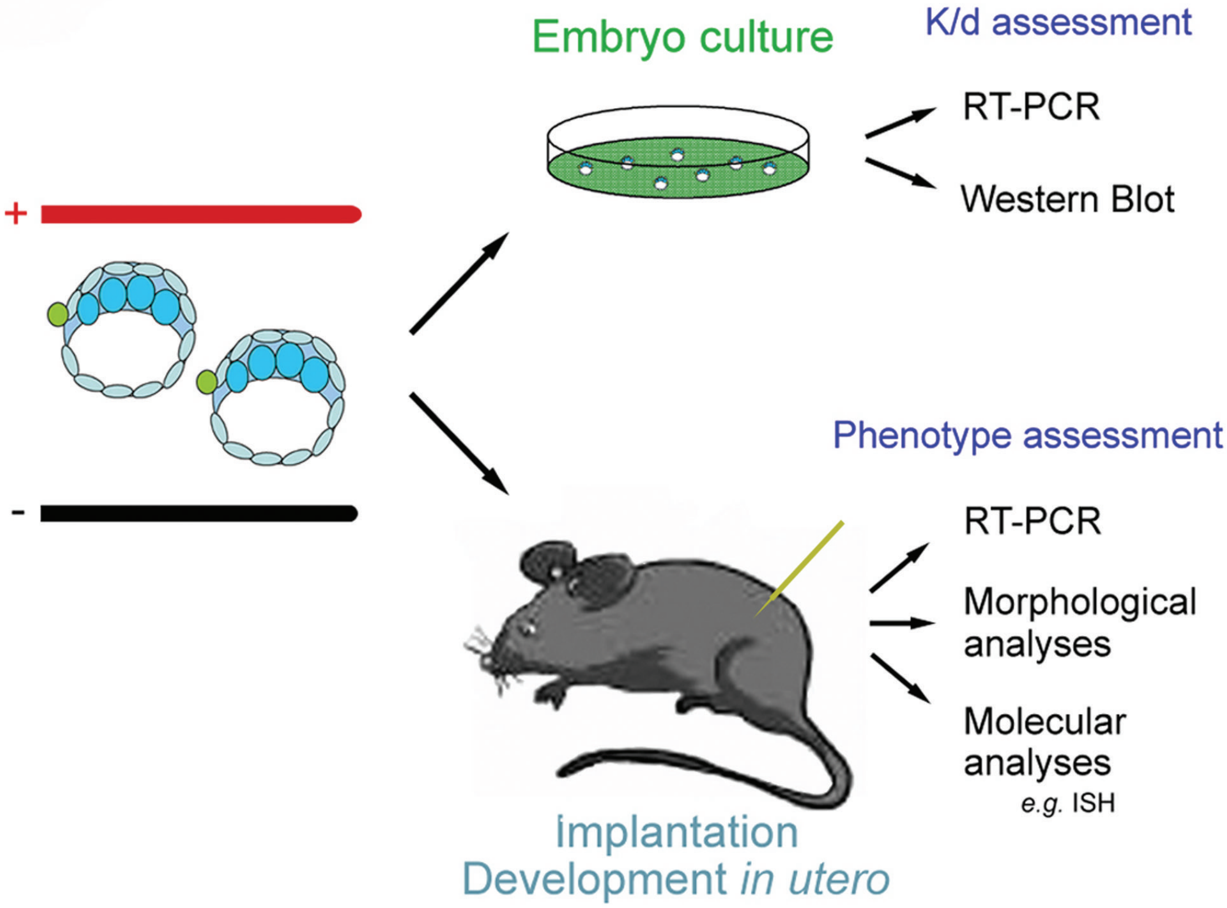

Method for RNAi in peri-implantation development

Supplementary Table 1

	Marker Gene														
	Brachyury			***Eomes***			***Cer-l***			***Lefty-1***			***Lhx1***		
	Group 1		Group 2	Group 1		Group 2	Group 1		Group 2	Group 1		Group 2	Group 1		Group 2
Emb Day	MN	DA	Df	MN	DA	Df	MN	DA	Df	MN	DA	Df	MN	DA	Df
E6.5	6	2	2	5	0	2	6	3	2	1	2	2	2	1	1
E7.5	7	1	3	4	0	3	3	4	3	1	2	4	1	2	2
Totals															
E6.5	8 / 15		2 / 2	5 / 9		2 / 2	9 / 16		2 / 2	3 / 6		2 / 2	3 / 5		1 / 1
E7.5	8 / 16		3 / 3	4 / 7		3 / 3	7 / 15		3 / 3	3 / 5		4 / 4	3 / 6		2 / 2
Total (%)	16 / 31 (52%)		5 / 5 (100%)	9 / 16 (56%)		5 / 5 (100%)	16 / 31 (52%)		5 / 5 (100%)	6 / 11 (55%)		6 / 6 (100%)	6 / 11 (55%)		3 / 3 (100%)
							SExp	DR	Abs						
E5.5 / E5.75	n / a			n / a			8	2	3	n / a			n / a		
Total (%)							13 / 16 (81%)								

Proportion of dsBmp4 RNA-electroporated embryos with defective expression of indicated marker genes.
